# Revealing the Therapeutic Potential of Stem Cells in Burn Healing: A Deeper Understanding of the Therapeutic Mechanisms of Epidermal Stem Cells and Mesenchymal Stem Cells

**DOI:** 10.1155/2024/1914585

**Published:** 2024-12-16

**Authors:** Jianyu Lu, Wei Zhang, Yushu Zhu, Pengfei Luo, Xirui Tong, Sujie Xie, Luofeng Jiang, Xinya Guo, Jie Huang, Minyi Gu, Xinran Ding, Shuyuan Xian, Runzhi Huang, Shizhao Ji, Zhaofan Xia

**Affiliations:** Department of Burn Surgery, The First Affiliated Hospital of Naval Medical University, Shanghai 200433, China

**Keywords:** bibliometrics, burns, epidermal stem cells, mesenchymal stem cells, therapeutic mechanisms

## Abstract

**Background:** Burns are a global public health issue and a major cause of disability and death around the world. Stem cells, which are the undifferentiated cells with the potential for indefinite proliferation and multilineage differentiation, have the ability to replace injured skin and facilitate the wound repair process through paracrine mechanisms. In light of this, the present study aims to conduct a bibliometric analysis in order to identify research hotspots of stem cell–related burns and assess global research tendencies.

**Methods:** To achieve this objective, we retrieved scientific publications on burns associated with stem cells covering the period from January 1, 1978, to October 13, 2022, from the Web of Science Core Collection (WoSCC). Bibliometric analyses, including production and collaboration analyses between countries, institutions, authors, and journals, as well as keyword and topic analyses, were conducted using the bibliometrix R package, CiteSpace, and VOSviewer.

**Results:** A total of 1648 burns associated with stem cell documents were published and listed on WOSCC. The most contributive country, institution, journal, and author were the United States, LV Prasad Eye Institute, *Burns*, and Scheffer C.G. Tseng, respectively. More importantly, combined with historical direct citation network, trend topic analysis, keyword co-occurrence network, and substantial literature analysis, we eventually summarized the research hotspots and frontiers on burns associated stem cell reasearch.

**Conclusion:** The present study obtained deep insight into the developing trends and research hotspots on burns associated with stem cells, which arouses growing concerns and implies increasing clinical implications. The mechanism and therapeutics of epidermal stem cells (ESCs) for burn wounds and the mechanism of mesenchymal stem cells (MSCs) and MSC-derived exosomes for burns wounds are two research hotspots in this field.

## 1. Introduction

Burns are a global public health issue and a major cause of disability and death around the world and are primarily caused by heat, electricity, radioactivity, radiation, friction, or chemical exposure [1]. According to the World Health Organization (WHO), there are 180,000 fatalities from burns annually (https://www.who.int/en/news-room/fact-sheets/detail/burns). In 2004, nearly 11 million people worldwide were severely burned and needed medical treatment. At present, the mortality rate of child burns in low- and middle-income countries is more than seven times that of high-income countries, while the mortality rate of burns in high-income countries has been decreasing. However, burn injuries are commonly associated with exaggerated immune and inflammatory responses, distribution shock, and metabolic changes, potentially leading to multiple organ failure, which is difficult to manage [2, 3]. Complications of burn injuries, such as infection, muscle and bone mass loss, poor wound healing, scarring, and psychological sequelae including posttraumatic stress disorder and depression, affect the physical and mental health seriously and diminish the quality of life of patients [4]. Furthermore, burns pose a major economic burden on families and society. Despite these challenges, current treatments for burns and trauma remain extremely limited due to a lack of comprehensive understanding of the cellular and molecular mechanisms underlying tissue repair and wound healing.

Optimal recovery from skin tissue injury depends on the patient's intrinsic biology and regenerative capacity. The ideal healing after burn injury is to fully restore and repair the original structure and function of the skin. In the last few years, stem cells have emerged as the potential strategy to achieve tissue regeneration and graceful healing of burn wounds as a new potential cellular therapy, with the development of biomedical engineering. Stem cells, which develop from three primitive embryonic layers (endoderm, mesoderm, and ectoderm), are undifferentiated cells with the potential to proliferate indefinitely and differentiate into a diverse spectrum of mature cells [5]. They can efficiently replace the injured tissue and complete the process of wound repair through paracrine mechanisms. However, terminal differentiation caused by pathological inflammation impairs stem cell function and depletes stem cell populations [6]. As a result, poor tissue regeneration and prognosis occur. It is noteworthy that stem cell therapy is successfully used in experimental in vivo studies to treat massive burns, hematologic malignancies, acute thrombocytopenia, graft-versus-host disease, and autoimmune diseases [7, 8]. In particular, the application of stem cells in burn healing shows great promise in regulating the inflammatory response, reducing fibrosis and infection, promoting the regeneration of the skin and its adnexa, accelerating wound healing and improving scar proliferation [9]. Proteomic analysis has revealed that stem cells secrete a variety of cytokines, chemokines, and growth factors that promote the proliferation and migration of keratinocytes, fibroblasts, and endothelial cells, aiding in the healing of burn wounds [10]. Furthermore, stem cell–derived exosomes have shown potential therapeutic significance for tissue injury by inducing regenerative phenotypes, maintaining stemness, inhibiting apoptosis, and immunomodulating [11]. Stem cells and their exosomes manipulate the burn wound environment by paracrine signaling through pathways including Wnt/B-catenin, PI3K/Akt, Hedgehog, and Notch, facilitating burn wound healing [12]. Notably, stem cells are also able to inhibit the activity of keloid fibroblasts and improve scar healing through paracrine signaling [13, 14]. Nevertheless, further research is required to gain insight into the involvement of stem cells in the development of burn wounds and the various cellular molecular mechanisms of treatment, as research on the use of stem cells in burn wounds is still at the preclinical stage. Therefore, it is essential to reach an understanding of the research trends or hotspots in order to advance burns associated with stem cell research.

Bibliometrics has already become a popular method for literature research. It can assist in quickly identifying research hotspots, developmental trajectories, and frontier topics in specific research areas based on statistical and visual analysis [15]. Recently, it has been heavily utilized in a number of research fields, including spinal cord injury, coronaviral illness, pancreatic cancer, and cardiovascular diseases [16–19]. These have made significant contributions to the discovery of the latest research focus and the guidance of clinical care. Since 2010, with the development of bioinformatics and scientific technology, the number of burns associated with stem cell studies has grown rapidly. There is no bibliometrics analysis currently available on burn associated with stem cell research. Hence, we compiled the scientific articles on burns associated with stem cell research since the 1978 from the Web of Science Core Collection (WoSCC) database. Subsequently, to recognize research hotspots and tendencies, the retrieved literature was further visualized and analyzed using the bibliometrix R package, VOSviewer, and CiteSpace [20–22]. In the present study, our work provided a list of countries, institutions, journals, and authors with the greatest publication and impact. In particular, the current knowledge structure, global status, developing trend, research hotspots, and future directions in burn associated with stem cells have been mapped.

## 2. Methods

### 2.1. Search Strategy

The search terms are chosen mainly based on the medical subject word list in the PubMed database, and the search strategy includes using the WOSCC database to search for relevant literature on October 13, 2022. The data retrieval algorithm was as follows: (((TS = burns) OR (TS = burn) OR (TS = burn injury) OR (TS = burn injuries) OR (TS = empyrosis) OR (TS = ambustion) OR (TS = scald) OR (TS = scalds) OR (TS = scalding) OR (TS = scalded)) AND ((TS = stem cell⁣^*∗*^))). Only research articles and reviews were retrieved. After eliminating literatures not meeting the language and types of artical requirements, we evaluated the titles and abstracts of the articles to determine which of the remaining literatures were needed to be included. All records and references were downloaded in TXT format; the raw data were listed in Supporting Information. To escape the deviation of frequent database updates, all searches and data extractions were performed on October 13. Furthermore, bibliometric tools were used to conduct further analysis.

### 2.2. Data Analysis

The analysis of burns associated with stem cells involved the use of various open-source tools, including bibliometrix R package, VOSviewer, and CiteSpace [20, 22], which were the same to our previous article [23]. To visualize the raw data obtained from WOSCC, the bibliometrix package (version 4.0.0) in R (version 4.2.2) was primarily utilized. This allowed for the examination of the average citation counts and annual scientific output in order to identify the trends of the field. Several bibliometric indicators, such as H-index, number of citations, and production, were then applied to assess the contributions and influence of journals, authors, institutions, and countries. Additionally, the collaboration networks of countries and authors were constructed to analyze intercountry and interauthor collaborations. Furthermore, the construction of two important networks, the historical direct citation and the keyword co-occurrence, revealed the main study content and related research areas of keywords [24]. Through an analysis of highly cited articles and high-frequency keywords, the research focus in this field were identified. Moreover, maps of two-dimensional thematic and trend topics were generated to gain insight into the research frontiers and future directions of burns associated with stem cells by analyzing key subject terms and their evolution over time.

## 3. Results

### 3.1. The Burns Associated With Stem Cells Are Arousing Increasing Concern

**Figure 1:**
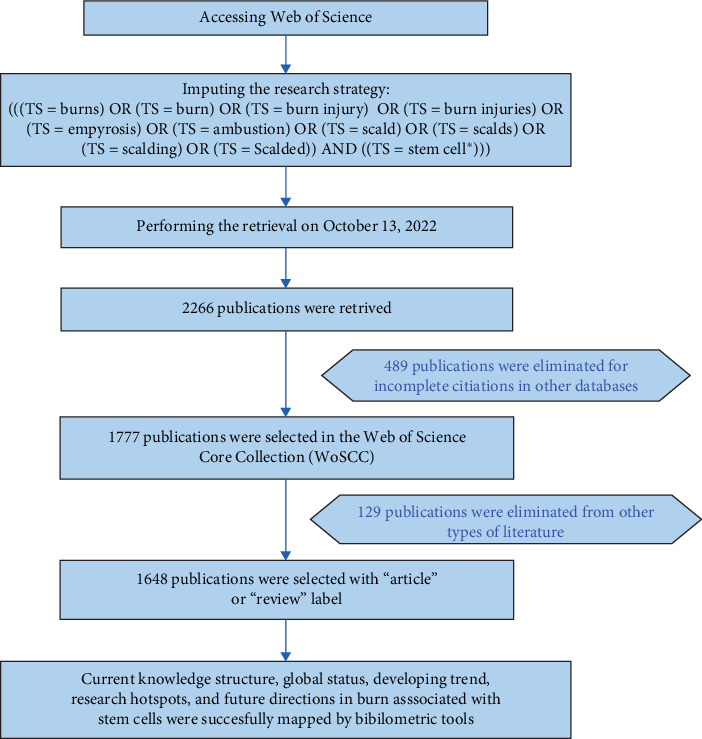
The retrieval process and exclusion and inclusion criteria.

From January 1, 1978, to October 13, 2022, based on the retrieval process shown in Figure 1, 1648 burns associated with stem cell documents were published and listed on WOSCC, and the total H-index is 114. Figure S1 presents the trend of annual scientific publications. The cumulative number of documents related to burns associated with stem cells has generally showed an upward trend with an annual growth rate of 10.62%. It has maintained a rapid growth especially since 2010 and has remained above 50 articles. This may be attributed to the evolution of bioinformatics technologies such as single-cell RNA sequencing, which has led researchers to subsequently study the characteristics, activation mechanisms, and functions of different stem cell subpopulations. In addition, the total number of citations for these documents is 58,543, with 35.52 average citations per documents. More importantly, according to Figure S1, in 1993 and 1997, two peaks in the annual article citations per year were recorded, which reveal a major breakthrough or possibly the crucial origin of burns associated with stem cell research by scholars in this period. This is also consistent with the results of our analysis of the literature.

### 3.2. Distribution Characteristics of Countries/Regions

**Figure 2:**
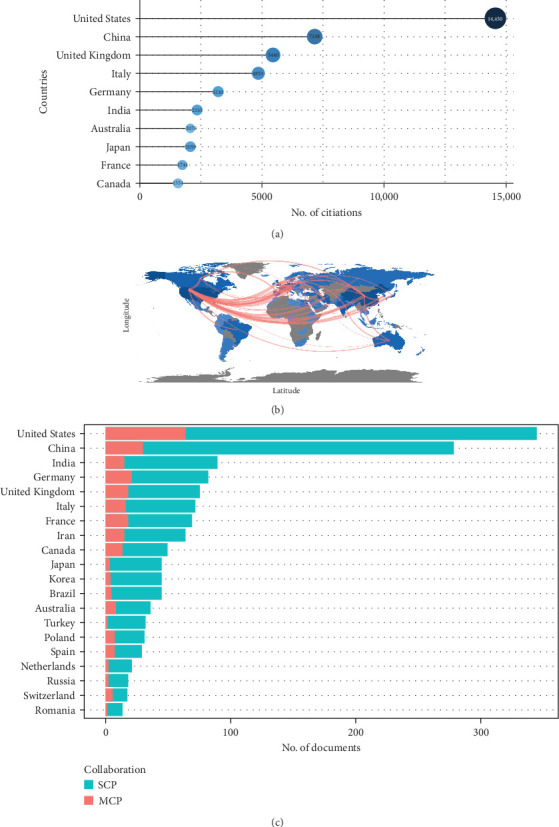
Central countries/regions of burns associated with stem cell research production and collaboration. The United States and China were the two most influential and contributing countries in burns associated with stem cell research. (A) The top 10 countries/regions of burns associated with stem cell research with the highest number of publications. (B) The top 10 countries/regions of burns associated with stem cell research with the highest number of citations. (C) Country/region production and collaboration world map of burns associated with stem cell research. MCP, multiple-country publication; SCP, single-county publication.

The map of the country scientific production (Figure S2) indicated the quantity and distribution of publications in various regions/countries around the world. We identified the top 10 highest producing countries, with 3669 publications (Figure 2A). The United States has the highest quantity of publications (*n* = 1025) with a total of 14,550 citations, ahead of China (881 records with 7148 citations) and Iran (305 records with less than 1550 citations). These data suggest that the United States and China had the largest publication volume and citation rates, making them the most prolific and influential countries in burns associated with stem cell research. The visualization country cooperation map further supported this point, in which the United States and China had the most critical central connection point and the closest connection observed between two countries (Figure 2B). The United States and China also ranked the highest in both single- and multiple-country collaborative articles (Table 1 and Figure 2C), indicating their extensive collaborations with other publishing countries. In contrast, other countries should strive to promote research progress and develop partnerships between countries.

Additionally, we ranked all institutions involved in burns associated with stem cells related on the quantity of publications. Among the highest 10 institutions (Figure S2), the LV Prasad Eye Institute published the most quantity of relevant publications (*n* = 98), ahead of the University of Texas Medical Branch (*n* = 53) and the University of Shahid Beheshti Medical Sciences (*n* = 52). Notably, almost half of the top 10 institutions with the most quantity of publications are affiliated with the United States, once again emphasizing the academic influence and contribution of the United States in this field. Therefore, we propose that the United States and China are the most contributing and influential countries in burns associated with stem cell research.

### 3.3. Distribution Characteristics of Journal

**Figure 3:**
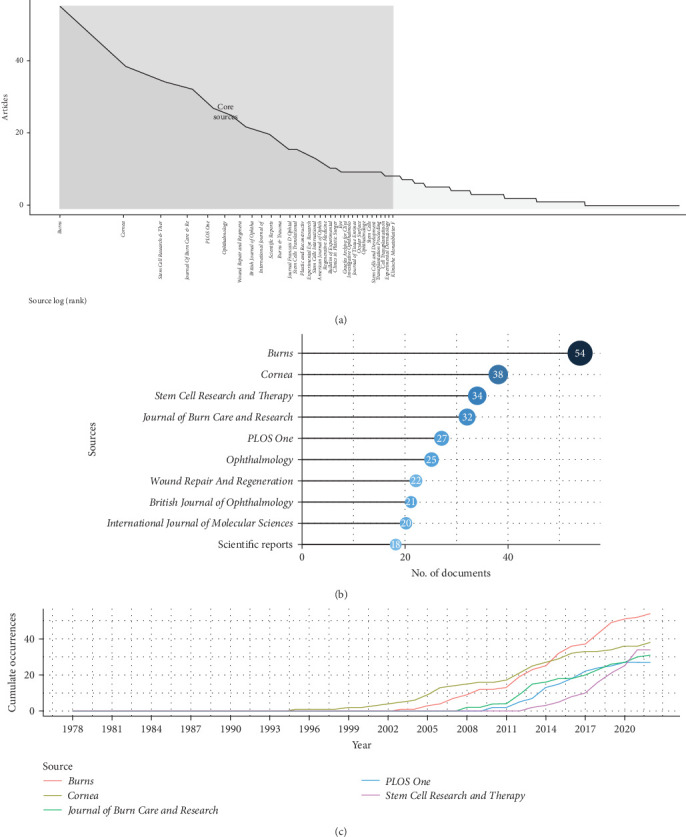
The *Journal of Wound Repair and Regeneration* was a critical pathway to access the research frontiers and crucial information of burns associated with stem cell research. (A) Core journals of burns associated with stem cell research based on Bradford's law. (B) The top 20 journals on burns associated with stem cell research with the highest number of publications. (C) The six highest yielding journals' growth of burns associated with stem cell research from 2000 to 2022.

There are 655 sources of publications on burns related to stem cells, such as journals and books. According to Bradford's law and quantity of publications, 33 journals with a high productivity were classified as core sources (Figure 3A) [25]. Among these, a total of 291 publications were published in the top 10 highest producing journals, which also accounted for the majority of citations with a total of 16,713 times (Figure 3B and Figure S2). Particularly, the academic journal *Burns* had the maximum number of articles (*n* = 54), ahead of *Cornea* (*n* = 54), *Stem Cell Research & Therapy* (*n* = 34), *Journal of Burn Care & Research* (*n* = 54), and *PLOS One* (*n* = 27). In terms of citations received, *Burns* received the highest number (*n* = 3089), followed by *Ophthalmology* (*n* = 1798), *Investigative Ophthalmology & Visual Science* (*n* = 1711), and *Cornea* (*n* = 1569). These high-production journals serve as invaluable sources of intellectual knowledge in the field of burns associated with stem cells. What is more, *Burns* has the highest number of productions and citations, indicating his notable influence in this area. Therefore, focusing on this journal allows for immediate access to the frontiers of researches and key information. Moreover, Figure 3C demonstrates the rapid increase in the productivity of top six highly prolific journals over time.

### 3.4. Distribution Characteristics of Author

Since 1978, more than 7395 authors have published on burns associated with stem cell research. According to Lotka's law, 80.2% of these authors have only published one article, while 163 authors have exceeded five publications. Among the authors, we examined the top 10 most productive ones who collectively accounted for 11.23% of all articles (Figure S3). Using the Biblioshiny software, we analyzed the top 10 most locally cited authors, the top 10 most productive authors, and the top 10 most locally impactful authors determined by the H-index (Figure S3). Tseng [26] had the highest number of publications (*n* = 21) with 495 total citations (TCs) and an H-index of 18. Following closely was Facon et al. [27] (*n* = 21) with 183 TCs. Interestingly, despite publishing relatively few articles, Corradini et al. [28] ranked second in terms of citation count and impact, with 477 TCs and an H-index of 14. This suggests that Pellegrini, De Luca, and Arsenijevic [29] may have contributed landmark works in the field. Analyzing the top author's production over time map (Figure S4), we found that Tseng [26] was an original and pioneering author in the field, publishing his masterpiece as early as 1995 and contributing significantly to its development. On the other hand, Facon et al. [27] emerged as one of the most active authors in the last decade, demonstrating a substantial number of meaningful publications. Additionally, based on the author's collaboration network, Facon et al. [27] appeared to have the closest collaborations with other authors (Figure S4).

### 3.5. The High-Frequency Keyword Analysis and Two Research Focus Stem from Keyword Co-Occurrence Analysis

**Figure 4:**
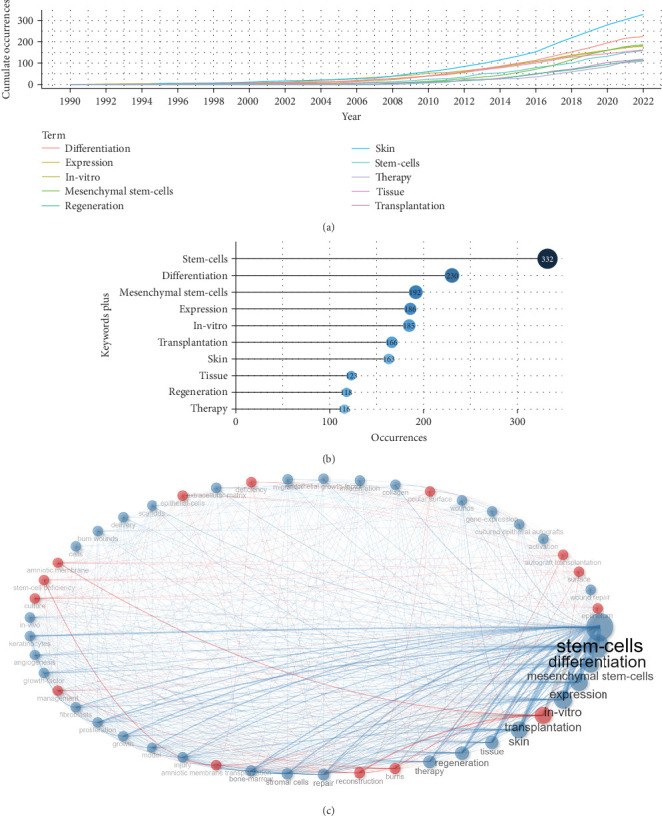
Analysis of high-frequency keywords and four research hotspots based on the keyword co-occurrence analysis. (A) Top 10 most frequent keyword growth of burns associated with stem cell research from 2000 to 2022. (B) Visualized word cloud map based on the top 50 most frequent keywords for burns associated with stem cell research. (C) Visualized keyword co-occurrence network for burns associated with stem cell research. Each node indicates a keyword, and the connecting lines between nodes denote the intimacy between keywords. The four clusters were red, blue, green, and purple.

Figure S5 was used to identify the top 50 high-frequency keywords associated with burns and stem cell research. These keywords serve as a condensed version of the article's essential content and are effective in identifying research hotspots and critical details [17]. The frequency of the top 10 most frequent keywords increases over time, as shown in Figure 4A. Interestingly, “expression” has consistently held the first place in terms of frequency of occurrence since 1996, especially after 2010. Figure 4B displays the top 10 most common keywords, with “stem-cells” having the most times (*n* = 332), ahead of “differentiation” (*n* = 230), “mesenchymal stem-cells” (*n* = 192), “expression” (*n* = 186), “in-vitro” (*n* = 185), “transplantation” (*n* = 166), “skin” (*n* = 163), “tissue” (*n* = 123), “regeneration” (*n* = 118), and “therapy” (*n* = 116). To establish keyword relationships, a keyword co-occurrence analysis was performed, resulting in the keyword clustering network(Figure 4C). In this figure, a keyword was represented by a node, with the size of node indicating popularity and lines indicating the intimacy between the keywords. This analysis provides valuable insights into keyword relationships and identifies important thematic areas within the dataset:

1. Cluster 1 (blue): The keywords “stem-cells” (*n* = 332), “differentiation” (*n* = 230), “expression” (*n* = 186), and “in-vitro” (*n* = 185) were found in this blue cluster. These keywords mainly displayed the mechanisms of stem cells in promoting the reparation of burn injury, particularly the mechanisms of molecular and cellular related to the differentiation potential of mesenchymal stem cells (MSCs).
2. Cluster 2 (red): The most common keywords in this red cluster are “transplantation” (*n* = 166), “burns” (*n* = 102), “reconstruction” (*n* = 101), and “amniotic membrane transplantation” (*n* = 81). This group of keywords largely focused on the transplantation of amniotic membrane for burn wound healing.

### 3.6. The Associations Between the Hotspots, the Historical Evolution, and the High-Impact Literature

**Figure 5:**
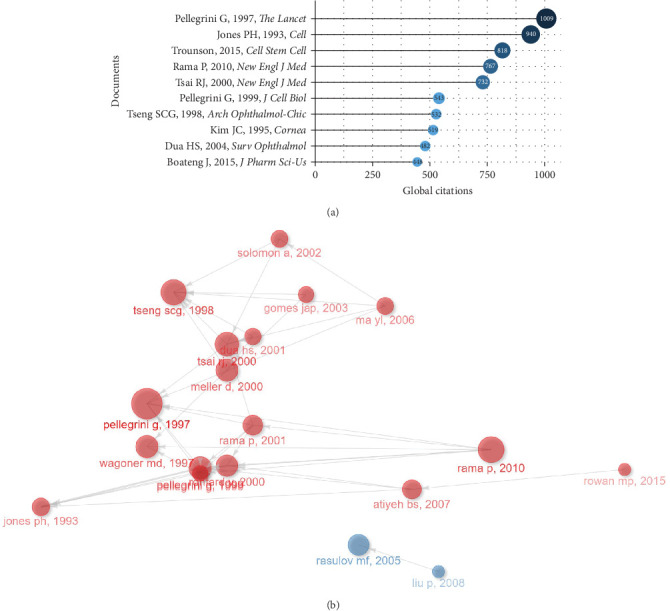
Relationship between high-impact literature and historical evolution and hotspots. (A) The top 20 most global cited documents of burns associated with stem cell research. (B) Visualized historical direct citation network based on the evolution trend of burns associated with stem cell research from 2000 to 2022. Each node represents a piece of literature, and the lines between the nodes indicate the citation relationships between publications.

Table 2 displayed the top 10 documents on burns associated with stem cells with the highest number of global citations, and 8 documents had more than 500 citations. Subsequently, Figure 5A and Table 3 summarized the top 10 most locally cited of the 1648 publications, along with their years of publication, journals, and authors. The article of Pellegrini et al. [24] titled “Long-term restoration of damaged corneal surfaces with autologous cultivated corneal epithelium,” published in 1997 in *The Lancet*, was the most locally cited article with 131 citations. He pioneered the use of autologous cultured corneal epithelium to repair damaged corneas, which is a possible breakthrough in research on repairing injured tissue through stem cell transplantation. Meanwhile, this validates our results in the previous authors' analysis. Additionally, the article “Limbal stem-cell therapy and long-term corneal regeneration” by Rama et al. [30] in 2010 from the New England Journal of Medicine had 90 citations, and the article “Amniotic membrane transplantation with or without limbal allografts for corneal surface reconstruction in patients with limbal stem cell deficiency” by Tseng [26] in 1998 from the *Archives of Ophthalmology* had 85 citations. These articles have validated the safety and efficacy of corneal limbal stem cells for the repair of injured corneas. More importantly, they have laid a reliable foundation for guiding the repair and reconstruction of burns-related injured tissues in clinical practice.

We created a historical direct citation network (Figure 5B) to deeply explore the major contents and advancements in stem cell and burn trauma studies. By identifying the relationships among highly cited publications, this network established connections between nodes representing literature and lines suggesting citation relationships between articles. Using the network, we categorized the articles into two clusters (Table 4), representing two significant research themes during their respective periods.

At that time, the first cluster (red) can be derived from 1993, when scholars represented by Jones and Watt [31] dedicated their studies to the techniques associated with the isolation, culture, and transplantation of stem cells. Their focus was primarily on the restoration of damaged tissues, particularly epidermal stem cells (ESCs) and corneal–limbal stem cells [24, 30, 32–34]. The efficient isolation of ESCs from the cultured epidermis was achieved through the differentiation in integrin function and expression. Additionally, the culture on fibrin matrices facilitated stem cell survival. Conversely, the second cluster (blue) papers discussed the safety and efficacy of MSC transplantation in the treatment of burn wounds. Both clinical and animal studies have indicated that the administration of MSCs accelerates wound epithelialization and healing compared to control groups, promoting angiogenesis [35, 36]. More importantly, the concentration of articles published in this period, from 1993 to 2001, coincides with the period in which the peak number of annual citations occurs, in particular, published in 1997 by Pellegrini et al. [24] on the repair of damaged corneas by autologous cultured corneal rim epithelium. It was cited by 6 articles within cluster 1, with 131 local citations (LCs) and 1009 TCs, ranking first. Therefore, we hypothesized that articles from this period may serve as both the pivotal origin and breakthrough for burns related to stem cell research. Additionally, these research themes might reflect the evolution of prominent areas of interest in this field.

### 3.7. Current Research Status of Diverse Hot Topics on Burns Associated With Stem Cells

**Figure 6:**
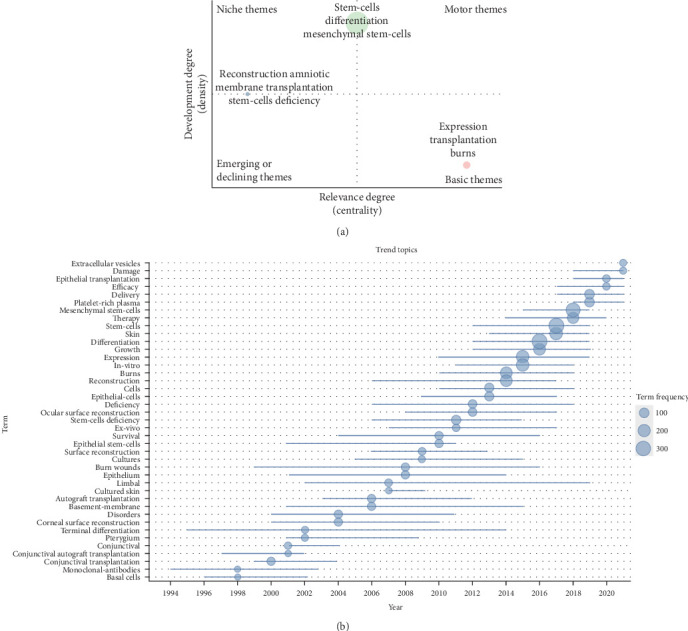
Exploring research status of various hot topics on burns associated with stem cells, sketching historical trajectories and revealing research frontiers. (A) Thematic map for burns associated with stem cell research. The horizontal coordinate refers to the relevance degree (centrality), and the vertical coordinate represents the development degree (density). Motor themes in the first quadrant represents core themes with high centrality and maturity, niche themes in the second quadrant represent isolated themes with increased maturity, the third quadrant represents emerging or declining themes with low centrality and high maturity, and basic themes in the fourth quadrant means popular themes with low maturity. (B) Trend topic map for burns associated with stem cell research. Showing trends in the occurrence of high-frequency keywords for burns associated with stem cell research.

A two-dimensional thematic map (Figure 6A) was constructed using centrality on the horizontal axis and density on the vertical axis. Centrality indicates the closeness between the theme and others, representing the core of the research area. Density indicates the level of development of a single theme, with higher values representing greater matureness. The green cluster, located at the junction of the motor theme and the niche theme on the vertical axis, represents a highly developed theme. Keywords for this cluster include “stem-cells,” “differentiation,” and “mesenchymal stem-cells,” which align with cluster 1 in the keyword co-occurrence analysis on the cellular and molecular mechanisms of MSCs in burn tissue repair. This theme has received longstanding scholarly attention and has yielded concrete results. However, further substantial groundbreaking research is needed. The blue theme, representing low centrality, is located at the junction of niche themes and emerging or declining themes on the horizontal axis. Keywords for this cluster include “reconstruction,” “amniotic membrane transplantation,” and “stem-cell deficiency.” This suggests that the use of amniotic membrane transplantation in ocular corneal rim stem cell defects and related mechanisms is gradually being neglected by scholars. The red cluster, located in the third quadrant of the base theme, indicates topical research that is not yet mature in development. Keywords for this cluster include “expression,” “transplantation,” and “burns.” Similar to cluster 1 in the keyword co-occurrence analysis, this theme focuses on the therapeutic efficacy and molecular mechanisms of ESC transplantation for burn wounds.

### 3.8. Outlining the Historical Track and Discovering the Frontiers of Researches Through Trend Topic Analysis

A visualization of top keywords over the past decades by a trend topic map was constructed (Figure 6B) to explore the historical development trajectory in this field with greater accuracy. It is important to note that the evolution of research topics related to burn trauma and stem cells is closely linked to the development of bioinformatics. In the early years, the keywords included “conjunctival autograft transplantation,” “corneal surface reconstruction,” and “autograft transplantation” which reflected the prioritization of saving lives and reducing complications due to the limited availability of technologies like high-throughput sequencing. However, with the introduction and widespread use of next-generation sequencing (NGS) technology in 2008 and the subsequent decrease in its price, the technologies of second-generation sequencing and single-cell sequencing became more prevalent [37]. This led scholars to focus on understanding the cellular and molecular mechanisms involved in stem cell differentiation and the pathophysiological process of injury repair in burn wounds. As a result, the keywords shifted to concepts like “expression,” “differentiation,” and “stem cells.” Furthermore, the rapid advancements in sequencing techniques such as metagenomics, 16S-rDNA-seq, and single-cell RNA sequencing allowed researchers to delve into the characteristics, activation mechanisms, and functions of different stem cell subpopulations. Consequently, recent keywords have been centered around “delivery,” “efficacy,” and “extracellular vesicles.”

## 4. Discussion

**Figure 7:**
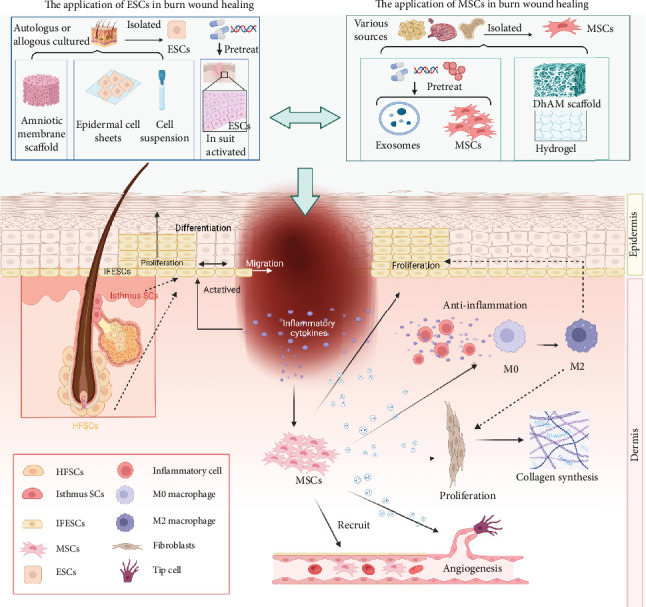
The molecular mechanisms and application of ESCs and MSCs in wound healing. ESCs are mainly distributed in the basal layer of the epidermis, the bulge area of the hair follicle, and the base of the sebaceous gland. After wound formation, ESCs are activated in response to inflammatory factors and undergo proliferation, migration, and differentiation. Activating the activity of ESCs in situ on the wound surface or through drugs, autoprotein, or gene editing to promote the increase of the original ESC activity of the wound surface and achieve the goal of accelerating the wound healing. MSCs play an important role in all stages of burn wound healing, including inflammation, angiogenesis, re-epithelialization, wound closure, and collagen remodeling, by secreting exsomes containing multiple functional proteins, mRNA, and microRNA. DhAM, acellular human amniotic membrane; ESCs, epidermal stem cells; HFSCs, hair follicle stem cells; IFESCs, induced pluripotent stem cells; MSCs, mesenchymal stem cells; SCs, stem cells.

This section will discuss the primary types of skin stem cells and their associated therapies to shed light on the three current areas of focus in burn-related stem cell research (Figure 7). There are several different kinds of stem cells in the skin, each with different functions. Examples of these include MSCs and ESCs [38]. Under normal circumstances, multipotent stem cells like MSCs and specialized stem cells like ESCs work together to maintain the equilibrium of the skin and regulate the healing process under normal circumstances [39]. The dermis can be rebuilt with the help of mesenchymal cells either from the surrounding undamaged skin or those recruited from it [40, 41]. Recent research indicates that myeloid cells may also have the potential to transform into mesenchymal cells and contribute to dermal reconstruction [42, 43]. Additionally, located in the basal layer, ESCs assist in epithelization by proliferating and differentiating into various types of keratinocytes, replacing damaged or defective cells [44]. This section will discuss the primary types of skin stem cells and their associated therapies to shed light on the three current areas of focus in burn-related stem cell research (Figure 7).

### 4.1. Overviews on Burns Associated With Stem Cell Research

A comprehensive analysis of burns associated with stem cell studies was conducted in the current study using an information visualization approach based on bibliometrix, VOSviewer, and CiteSpace software. Between January 1, 1978, and October 13, 2022, 1648 relevant publications were retrieved totally. The results suggested the study on burns associated with stem cells has been steadily growing, with a rapid increase in publications following the explosion of bioinformatics in 2010. This indicates a significant interest among researchers in burns associated with stem cells, given its clinical implications and developmental potential. The United States was the largest contributor and influential country in this field, followed by China and Iran. *Cornea*, *Stem Cell Research & Therapy*, and *Burns* were the top three journals with the highest number of publications in burns associated with stem cell research. We hypothesized that Tseng [26], Facon et al. [27], and Pellegrini and De Luca [45] were the top three most influential and contributing authors in the field, with clinically significant and fundamental potential for experimental development. Notably, Pellegrini et al. [46] may have published some landmark works in this area, pioneering meaningful research.

Keyword analysis is one of the indispensable parts of bibliometric analysis as it captures the typical content and topic of a specific article, representing a research hotspot. In this study, we conducted a comprehensive bibliometric analysis to investigate the research pattern and future research direction in burns associated with stem cells. Firstly, we examined the high-frequency keywords and highly cited documents. Subsequently, we analyzed the keyword co-occurrence network, historical direct citation network, topic map analysis, and substantial literature analysis. Through this process, we identified two research hotspots: the mechanism and therapeutic of ESCs for burn wounds and the mechanism of MSCs and MSC-derived exosomes for burn wounds. Moreover, by combining historical direct citation networks, thematic maps, trending thematic maps, and literature review analysis, we outlined the historical trajectory of this field and predicted future research directions based on hotspots and clinical experience. Our hypothesis suggests that the position of stem cell extracellular vesicles in injury and regeneration, particularly their function as novel drug delivery vehicles, could be a frontier research direction. Recent studies have shown that extracellular vesicle-loaded hydrogels hold promise as an effective therapeutic strategy for burns repair [47]. Overall, we speculate that the exploration of the role of stem cell extracellular vesicles in injury and regeneration could be a significant area of research in burns associated with stem cells.

### 4.2. Mechanisms and Therapeutics of ESCs in Burn Wound Recovery

#### 4.2.1. Mechanisms of ESCs in Burn Wound Healing

ESCs are a group of cells with unlimited proliferative capacity that can proliferate and differentiate into various functional cells in the epidermis. They are mainly distributed in the basal layer of the epidermis, the bulge area of the hair follicle (HF), and the base of the sebaceous gland. ESCs at the basal layer of the epidermis mostly differentiate into other epidermal cells [31, 48] They express high levels of α6 and β1 integrins, melanoma-associated chondroitin sulfate proteoglycan, and Leu-rich repeats and immunoglobulin-like domain 1 (LRIG1) [49, 50]. These ESCs replenish the basal layer and produce nonproliferative, transcriptionally active spinous and stratum granulosum and the outer layers of terminally differentiated stratum corneum ultimately [51, 52]. Hair follicle stem cells (HFSCs) are mostly distributed in bulges and have specific bulge markers, such as CD34 [38], G-protein-coupled receptor 5 (LGR5) [53], keratin 15/19 (KRT15/19) [54], and so on. According to the report, HFSCs have high clonality and produce all lineages of HFs and sebaceous glands when cultured in vitro [55]. In addition, stem cells distributed in different positions of sweat glands express different markers. Stem cells located at the junction between HFs and PR/SET domain 1 (PRDM1) and glands express LRIG1 and LGR6, while stem cells expressing GATA-binding protein6 (GATA6) are located at the entrance of glands [56, 57]. In general, all ESCs are conducive to maintaining epidermal homeostasis and promoting wound healing.

In steady state, ESCs maintain self-renewal and ensure the normal tissue structure of the skin by differentiating into different types of keratinocytes through asymmetric division. This dynamic balance is necessary to compensate for the continuous shedding of outer cells [58]. However, in partial thickness burn wounds, ESCs in residual epidermal tissue or appendages respond by rapidly increasing cell numbers through symmetrical division, reducing the proportion of differentiated cells to compensate for the damage and loss of cells during wound healing [59]. The response of ESCs to injury depends not only on their specific niche but also on their distance from the wound [60]. ESCs near the wound are activated and recruited, including a variety of ESCs. HFSC, when involved in repair, loses specific surface markers and adopts a phenotype similar to ESCs at the basal layer of the epidermis, although these effects are temporary and disappear after a few weeks. The effectiveness of this recruitment process has been confirmed through the delayed regeneration of HFSCs in HF-deficient mice with incision wounds, indicating the regeneration ability of HFSCs [61]. Conversely, the ESCs at the basal layer of the epidermis and those in the upper isthmus/funnel can maintain their ability to participate in repair for a long time [62]. While ESCs continue to proliferate, cells in the basal layer and upper basal layer migrate in the form of cell slices, with cells closer to the front exhibiting maximum migration speed [63]. There is a positive feedback mechanism between cell proliferation and migration, where cell migration causes basal cells to divide in the same direction, and enhanced proliferation promotes migration, pushing the leading edge toward the center of the wound [64]. Furthermore, migrating epidermal cells acquire epithelial–mesenchymal transformation (EMT) characteristics, including increased expression of EMT markers, decreased expression of cell adhesion molecules, and enhanced movement, which are essential for wound healing [65]. Interestingly, inflammatory cells can activate ESCs to a certain extent, promoting re-epithelialization and wound-induced HF regeneration [66]. Studies have shown that ESCs undergo dedifferentiation and acquire stem cell characteristics due to the influence of the inflammatory environment [67]. Specifically, the expression of Wnt7b and Wnt10a on skin-resident macrophages is increased, inducing ESCs to transition from a resting stage to a proliferating stage [68]. Additionally, another study found that macrophage apoptosis activates the Wnt/β-catenin signal pathway in ESCs, further promoting their proliferation [67].These behaviors are regulated by multiple signal pathways. The interaction between Notch and Wnt pathways, mediated by jagged1, plays a crucial role in wound healing. By targeting Hes1 and c-Myc, Notch and Wnt signals affect the migration, proliferation, and differentiation of ESCs and promote HF regeneration, ultimately enhancing wound healing [69]. Sustained activation of LIM homeobox 2 (LHX2) signaling in ESCs is essential for differentiation to occur, involving upregulation of transcription factor 4 (TCF4) and sex-determining region Y box protein 9 (SOX9), both of which promote wound re-epithelialization [70].

#### 4.2.2. Therapeutics of ESCs in Burn Wound Treatment

The application direction of ESCs in wound management mainly includes two aspects. One is to activate the activity of ESCs in situ on the wound surface. This method involves activating the relevant signal pathway through drugs, autoprotein, or gene editing to promote the increase of the original ESC activity of the wound surface, thus accelerating wound healing [71, 72]. The second method mainly includes cultured epidermal autografts (CEAs); that is, cells isolated from skin biopsies are propagated and cultured on biomaterials [73]. EpiDex, a commercially available self-produced product from HFSC, has been used in clinical practice since 2004. Long-term clinical trials have shown that EpiDex is particularly effective for chronic wounds with granulation but no longer epithelialized. These trials have also demonstrated that EpiDex can induce complete wound healing within a 9-month monitoring period [74]. In addition, the preparation of autologous ESC suspension that can be directly sprayed on the wound or the subcutaneous injection of allogeneic ESCs can help to shorten the wound healing time [75]. Researchers have also utilized cultured epidermal cell (CES) sheets in order to treat skin injuries [32]. Studies have demonstrated that CES enriched with ESCs can further improve wound healing, provide long-term regeneration, and prevent hypertrophic scar formation [76]. In vitro culture technology advancements have facilitated the expansion of a large number of ESCs. Human amniotic membrane has been used to prepare dermal scaffolds with intact basement membrane and good biological stability to allow for rapid expansion and transplantation of EPSCs [77]. In further development, the emerging technology of three-dimensional (3D) bioprinting has enabled the generation of customized composite skin products, providing a microenvironmental ecological niche structure for the growth and maintenance of stem cells [78].

In a word, the use of ESCs is expected to be an effective tissue repair strategy, thanks to their proliferative activity and differentiation potential. Moreover, with the advent of novel technologies such as 3D printing and tissue engineering, there is potential for using ESCs as seed cells to construct artificial composite skin. This emerging field of research may pave the way for future advancements in tissue engineering.

### 4.3. Mechanisms and Therapeutics of MSCs and MSC-Derived Exosomes in Burn Wound Recovery

#### 4.3.1. Mechanisms of MSCs and MSC-Derived Exosomes in Burn Wound Healing

After burns, a proportion of MSCs remain in the residual dermis of the injured surface and participate in tissue repair. This was also demonstrated in studies where MSCs were successfully extracted from postsurgical waste skin from burn wounds and applied to excised wounds in animals to demonstrate their ability to promote healing [79]. At the same time, tissue damage upregulated inflammatory factors such as tumor necrosis factor-α (TNF-α), interleukin-1β (IL-1β), interferon gamma (IFN-γ), IL-6, and IL-12 [80]. Stem cell mobilization is activated by stromal cell–derived factor-1 (SDF-1) and chemokine C-X-C motif receptor 4 (CXCR4), and they are recruited to the wound surface to participate in repair [81, 82]. When these cells nest at the site of injury, they exert their biological effects mainly through paracrine mechanisms, secreting bioactive factors that act as immunomodulatory and trophic regenerators, as if they were performing tissue repair in situ [83].

During the inflammatory phase of burns, MSCs are capable of immunomodulation and are able to regulate both innate and adaptive immune responses [84, 85]. MSC-derived exosomes, primarily expressed through bioactive molecules, play a significant role in suppressing the inflammatory response. These extracellular vesicles promote the transformation of macrophages into M2 phenotype and contribute to the regeneration of skin tissue [86]. In addition, MSCs have a suppressive effect on the proliferation of activated helper T cells (Th cells), resulting in reduced secretion of IL-17 and IFN-γ by Th1 and Th17 cells, respectively. Simultaneously, the production of IL-4 secreted by Th cells increased, indicating that T cells differentiated from proinflammatory to anti-inflammatory phenotypes [87, 88]. Furthermore, MSC-derived exosomes improve the inflammatory microenvironment by downregulating chemokine proinflammatory enzymes and cytokines (such as cyclooxygenase-2, inducible nitric oxide synthase, TNF, and monocyte chemotactic protein-1) while also upregulating anti-inflammatory factors such as IL-10 [89–92].

In the proliferative phase of wound healing, the activation and migration of skin constituent cells such as fibroblasts and keratinocytes occur [93]. Initially, MSCs manipulate macrophages to recruit keratinocytes and fibroblasts [94]. Subsequently, MSCs utilize exosomes to regulate the expression of growth factors and associated genes, thereby promoting the activity of the recruited cells [95]. Hu et al. [96] found that exosomes from MSCs can be taken up by fibroblasts and stimulate cell proliferation and collagen synthesis by upregulating the expression of related genes such as cell cycle protein-1 and N-calmodulin. Similarly, exosomes can stimulate the migration and proliferation of keratinocytes, possibly by upregulating proliferation markers such as cell cycle protein A2 and cell cycle protein D2 [97]. In a study using a rat skin burn model, it was found that exosomes containing Wnt4 facilitated the movement of β-linked proteins into the nucleus, leading to increased proliferation, migration, and wound epithelialization of keratinocytes [12]. Another effect of MSCs in wound healing is angiogenesis, which can be induced by MSC-derived exosomes exposed to neonatal mouse serum [98]. Additionally, exosomal delivery of miRNAs (let-7f, miR-30b, miR-30c, and miR-424) derived from MSCs can promote angiogenesis and vascular development in endothelial cells [99, 100].

In the final stage of wound healing, wound remodeling is strictly controlled to maintain the balance between collagen degradation and synthesis. MSCs, with their ability to enhance specific extracellular matrix (ECM) events, play a crucial role in healing wounds [101]. Evidence from a study confirms that the conditioned medium of MSCs promotes the secretion of collagen, elastin, and fibronectin by fibroblasts while inhibiting the expression of matrix metalloproteinase-1 [102]. Additionally, MSC-derived exosomes have been found to stimulate collagen synthesis in the early stage of wound healing but reduce collagen expression and scar formation during the later stage [103]. The exosomes also contain bioactive proteins and miRNAs that effectively inhibit scar formation. For instance, Fang et al. [104] discovered that MSCs derived from the umbilical cord exhibit high levels of multiple miRNAs (miR-21, miR-23a, miR-125b, and miR-145), which inhibit the TGF-β/SMAD2 pathway, thereby suppressing the formation of myofibroblasts.

To conclude, MSCs play a crucial role in all stages of burn wound healing—inflammation, angiogenesis, re-epithelialization, wound closure, and collagen remodeling—owing to their secretion of exosomes that contain multiple functional proteins, mRNA, and microRNA.

#### 4.3.2. Therapeutics of MSCs and MSC-Derived Exosomes in Burn Wound Treatment

The use of MSCs in the treatment of burn wounds consists of two main categories: the direct delivery of stem cells and their exosomes and other derivatives to the wound [105] and the combination of stem cells and their exosomes and other derivatives with tissue engineering or drugs [106]. However, the delivery of direct stem cells in the first type of approach has several limitations. Difficulties in tissue targeting and high cell attrition rates during delivery are some of the challenges. Moreover, the unfavorable wound environment results in low cell survival, additional cell damage, and lack of cell–ECM attachment [107]. To address these limitations, an alternative approach is to use stem cell secretory groups, such as conditioned medium or exosomes, to replace the cell itself [108]. MSC-derived exosomes, as a novel type of cell-free therapy, are considered safe, efficient, and easily prepared [92]. They overcome problems associated with cell transplantation, such as infection transmission, immune compatibility, complex material storage, and tumorigenicity [109]. Many animal experiments have confirmed the positive role of MSC secretion on burn wounds [110]. However, there is a limited number of clinical trials on cell-free treatment of burns, which require further validation.

In recent years, pretreatment, coculture, and genetic modification have emerged as methods to enhance the regenerative activity of MSCs. For example, a study demonstrated that the combination of MSC and platelet-rich plasma (PRP) improved the wound healing rate by promoting epithelial regeneration and regulating the inflammatory process [111]. Another study showed that human adipose-derived stem cells (ADSCs) expressing CA-Akt and v-myc exhibited higher proliferative potential and increased secretion levels of vascular endothelial growth factor (VEGF), leading to accelerated wound healing [112].

The combination of tissue scaffolds and stem cells has been a focus of research in tissue engineering. Different substitutes, such as scaffolds, hydrogels, and other natural or synthetic skin substitutes, have been explored as a means to create a suitable environment for stem cells. These substitutes not only provide nutrients [113] and facilitate gas exchange for the stem cells but also serve as an ECM for optimal cell adhesion [114, 115]. In 2014, in order to find an alternative to split-thickness autologous skin transplantation, the researchers introduced the use of MSCs on nanofibers, showing that this scaffold enhanced MSC function and facilitated healing [116]. Various scaffolds combined with MSCs, including hydrogel [117], fibrin matrix [118], and collagen [119], have shown promising results in burn wound healing. However, it is important to note that the combined use of stem cells and scaffolds is limited by design defects such as low mechanical strength, difficult operation, and potential immune rejection [120]. Furthermore, the timely degradation of the scaffold after implantation can have different effects on the release of biological factors from stem cells and the growth of new tissue [121].

On this basis, improving the characteristics of the scaffold to better adapt to the wound surface and assist stem cells is the main research direction for expanding the scope of stem cell therapy. With better delivery tools, the activity of stem cells and their secretions can be enhanced. A study found that acellular human amniotic membrane (DhAM) can be used as a scaffold for MSCs, offering advantages like low cost, high availability, and high safety compared to synthetic scaffolds. Furthermore, combining DhAM and synthetic scaffolds can regulate inflammation and promote faster epithelialization, suggesting DhAM's effectiveness and safety as a burn treatment in future clinical applications [122].

Our study has certain limitations that need to be acknowledged. Firstly, we solely relied on the publications gathered from the WoS Core Collection, which may have led to an incomplete compilation of all pertinent studies related to burns associated with stem cells research. Secondly, we confined our analysis to publications dated between January 1, 1978, and October 13, 2022, potentially excluding recent research findings. Nonetheless, despite these limitations, our study offers a comprehensive examination of the global status and research advancements pertaining to burns associated with stem cells.

To sum up, achieving rapid and effective burn wound healing in traditional burn management is challenging. Different strategies have been applied to improve the efficiency of various stem cells which have a broad application prospect in burn treatment. However, most of the studies at this stage are still limited to preclinical or small clinical studies. These studies lack large-scale and standardized clinical research data to confirm the clinical effectiveness of stem cell treatment. Furthermore, for future development of stem cell therapy, the optimization and standardization of parameters such as route of administration cell source, dose, and time are necessary. In addition, the transformation from basic research to large-scale stem cell expansion requires the expansion of stem cells on a large scale. This expansion necessitates the standardization of all parameters, including cell source, harvest method, cell inoculation density, and culture technology and equipment [123]. Besides, potential damage to cells such as oxidation and mechanical stress may occur during cell culture, leading to mutation and aging [124]. Hence, it is crucial to develop methods for large-scale expansion of stem cells while simultaneously avoiding potential cell damage. Based on this foundation, the combination of different types of stem cells and different combinations of stem cells with tissue engineering can be explored, offering new possibilities for stem cell therapy.

## 5. Conclusion

An in-depth insight into the current knowledge structure, research hotspots, development trends, and future directions of research investigating burns associated with stem cells was performed in the present study from a bibliometric perspective. The study reveals that research on burns associated with stem cells has experienced rapid growth, particularly since 2010. Two research hotspots in this field, namely, the mechanism and therapeutic potential of ESCs for burn wounds and the mechanism of MSCs and MSC-derived exosomes for burn wounds, have been identified. Further studies in these directions could enhance our understanding of the cell and molecular events of stem cells in treating burn wounds. Additionally, these results lay a strong foundation for identifying potential therapeutic targets for clinical conversion.

## Figures and Tables

**Table 1:** The top 10 most productive countries/regions of burns associated with stem cell research.

Rank	Country	Publications	Proportion of publications (%)	SCP	MCP	Proportion of MCP (%)
1	United States	345	20.90	281	64	18.60
2	China	279	16.90	250	29	10.40
3	India	90	5.50	75	15	16.70
4	Germany	82	5.00	62	20	24.40
5	United Kingdom	75	4.60	57	18	24.00
6	Italy	72	4.40	56	16	22.20
7	France	70	4.20	53	17	24.30
8	Iran	64	3.90	49	15	23.40
9	Canada	50	3.00	37	13	26.00
10	Japan	45	2.70	42	3	6.70

Abbreviations: MCP, multiple-country publication; SCP, single-country publication.

**Table 2:** The top 10 most global cited documents of burns associated with stem cell research.

Rank	Title	Journal	Author	Year	LCs	GCs	LC/GC ratio (%)
1	Long-term restoration of damaged corneal surfaces with autologous cultivated corneal epithelium	*The Lancet*	Pellegrini G	1997	131	1009	12.98

2	Limbal stem-cell therapy and long-term corneal regeneration	*New Engl J Med*	Rama P	2010	90	767	11.73

3	Amniotic membrane transplantation with or without limbal allografts for corneal surface reconstruction in patients with limbal stem cell deficiency	*Arch Ophthalmol-Chic*	Tseng SCG	1998	85	532	15.98

4	Reconstruction of damaged corneas by transplantation of autologous limbal epithelial cells	*New Engl J Med*	Tsai RJ	2000	75	732	10.25

5	The control of epidermal stem cells (holoclones) in the treatment of massive full-thickness burns with autologous keratinocytes cultured on fibrin	*Transplantation*	Pellegrini G	1999	68	278	24.46

6	Chemical injuries of the eye: current concepts in pathophysiology and therapy	*Surv Ophthalmol*	Wagoner MD	1997	66	343	19.24

7	Amniotic membrane transplantation for acute chemical or thermal burns	*Ophthalmology*	Meller D	2000	65	293	22.18

8	Long-term regeneration of human epidermis on third degree burns transplanted with autologous cultured epithelium grown on a fibrin matrix	*Transplantation*	Ronfard V	2000	62	242	25.62

9	Cytologic evidence of corneal diseases with limbal stem cell deficiency	*Ophthalmology*	Puangsricharern V	1995	60	372	16.13

10	First experience of the use bone marrow mesenchymal stem cells for the treatment of a patient with deep skin burns	*B Exp Biol Med+*	Rasulov MF	2005	59	132	44.7

Abbreviations: GCs, global citations; LCs, local citations.

**Table 3:** The top 10 most local cited documents on burns associated with stem cell research.

Rank	Title	Journal	Author	Year	LCs	GCs	LC/GC ratio (%)
1	Long-term restoration of damaged corneal surfaces with autologous cultivated corneal epithelium	*The Lancet*	Pellegrini G	1997	131	1009	12.98

2	Limbal stem-cell therapy and long-term corneal regeneration	*New Engl J Med*	Rama P	2010	90	767	11.73

3	Amniotic membrane transplantation with or without limbal allografts for corneal surface reconstruction in patients with limbal stem cell deficiency	*Arch Ophthalmol-Chic*	Tseng SCG	1998	85	532	15.98

4	Reconstruction of damaged corneas by transplantation of autologous limbal epithelial cells	*New Engl J Med*	Tsai RJ	2000	75	732	10.25

5	The control of epidermal stem cells (holoclones) in the treatment of massive full-thickness burns with autologous keratinocytes cultured on fibrin	Transplantation	Pellegrini G	1999	68	278	24.46

6	Chemical injuries of the eye: current concepts in pathophysiology and therapy	*Surv Ophthalmol*	Wagoner MD	1997	66	343	19.24

7	Amniotic membrane transplantation for acute chemical or thermal burns	*Ophthalmology*	Meller D	2000	65	293	22.18

8	Long-term regeneration of human epidermis on third degree burns transplanted with autologous cultured epithelium grown on a fibrin matrix	*Transplantation*	Ronfard V	2000	62	242	25.62

9	Cytologic evidence of corneal diseases with limbal stem cell deficiency	*Ophthalmology*	Puangsricharern V	1995	60	372	16.13

10	First experience of the use bone marrow mesenchymal stem cells for the treatment of a patient with deep skin burns	*B Exp Biol Med+*	Rasulov MF	2005	59	132	44.7

Abbreviations: GCs, global citations; LCs, local citations.

**Table 4:** Two clusters of historical direct citation network. Each node represents a piece of literature, and the connecting lines between nodes mean that later ones cite earlier articles.

Rank	Title	Author	Jounal	Year	LCs	GCs	Cluster
1	Separation of human epidermal stem cells from transit amplifying cells on the basis of differences in integrin function and expression	Jones PH	*Cell*	1993	46	940	1
2	Long-term restoration of damaged corneal surfaces with autologous cultivated corneal epithelium	Pellegrini G	*The Lancet*	1997	131	1,009	1
3	Chemical injuries of the eye: current concepts in pathophysiology and therapy	Wagoner MD	*Surv Ophthalmol*	1997	66	343	1
4	Amniotic membrane transplantation with or without limbal allografts for corneal surface reconstruction in patients with limbal stem cell deficiency	Tseng SCG	*Arch Ophthalmol-Chic*	1998	85	532	1
5	The control of epidermal stem cells (holoclones) in the treatment of massive full-thickness burns with autologous keratinocytes cultured on fibrin	Pellegrini G	*Transplantation*	1999	68	278	1
6	Location and clonal analysis of stem cells and their differentiated progeny in the human ocular surface	Pellegrini G	*J Cell Biol*	1999	41	543	1
7	Long-term regeneration of human epidermis on third degree burns transplanted with autologous cultured epithelium grown on a fibrin matrix	Ronfard V	*Transplantation*	2000	62	242	1
8	Reconstruction of damaged corneas by transplantation of autologous limbal epithelial cells	Tsai RJ	*New Engl J Med*	2000	75	732	1
9	Amniotic membrane transplantation for acute chemical or thermal burns	Meller D	*Ophthalmology*	2000	65	293	1
10	Autologous fibrin-cultured limbal stem cells permanently restore the corneal surface of patients with total limbal stem cell deficiency	Rama P	*Transplantation*	2001	54	390	1
11	A new classification of ocular surface burns	Dua HS	*Brit J Ophthalmol*	2001	43	159	1
12	Long-term outcome of keratolimbal allograft with or without penetrating keratoplasty for total limbal stem cell deficiency	Solomon A	*Ophthalmology*	2002	44	223	1
13	Amniotic membrane transplantation for partial and total limbal stem cell deficiency secondary to chemical burn	Gomes JAP	*Ophthalmology*	2003	42	133	1
14	Reconstruction of chemically burned rat corneal surface by bone marrow-derived human mesenchymal stem cells	Ma YL	*Stem Cells*	2006	44	254	1
15	Cultured epithelial autograft (CEA) in burn treatment: three decades later	Atiyeh BS	*Burns*	2007	51	185	1
16	Limbal stem-cell therapy and long-term corneal regeneration	Rama P	*New Engl J Med*	2010	90	767	1
17	Burn wound healing and treatment: review and advancements	Rowan MP	*Crit Care*	2015	38	403	1
18	First experience in the use of bone marrow mesenchymal stem cells for the treatment of a patient with deep skin burns	Rasulov MF	*B Exp Biol Med*+	2005	59	132	2
19	Tissue-engineered skin containing mesenchymal stem cells improves burn wounds	Liu P	*Artif Organs*	2008	38	105	2

Abbreviations: GCs, global citations; LCs, local citations.

## Data Availability

The datasets generated and/or analyzed during the current study are available in the supplementary materials.

## Supporting Information

Supporting Information Figure S1. The growth of burns associated with stem cells is arousing increasing concern. Figure S2. Supporting information for Figure 2 and Figure 3. Figure S3. Primary authors of burns associated with stem cell research production. Woo Kyung Kim and Jonathan A. Garlick were the two most influential and contributing authors in burns associated with stem cell research. Figure S4. Top authors' production over time and collaboration. Figure S5. Keywords analysis. Visualized tree map depends on the top 50 most frequent keywords for burns associated with stem cells research. The top 10 most frequent keywords for burns associated with stem cell research.
