# Supplementary material for: Revealing the Therapeutic Potential of Stem Cells in Burn Healing: A Deeper Understanding of the Therapeutic Mechanisms of Epidermal Stem Cells and Mesenchymal Stem Cells
Source: Stem Cells Int. 2024 Dec 16;2024:1914585. doi: 10.1155/2024/1914585 (PMC11666318; doi:10.1155/2024/1914585)
Supplement: Supporting Information — Figure S1. The growth of burns associated with stem cells is arousing increasing concern. Figure S2. Supporting information for Figure 2 and Figure 3. Figure S3. Primary authors of burns associated with stem cell research production. Woo Kyung Kim and Jonathan A. Garlick were the two most influential and contributing authors in burns associated with stem cell research. Figure S4. Top authors' production over time and collaboration. Figure S5. Keywords analysis. Visualized tree map depends on the top 50 most frequent keywords for burns associated with stem cells research. The top 10 most frequent keywords for burns associated with stem cell research. [file 1914585.f1.docx]

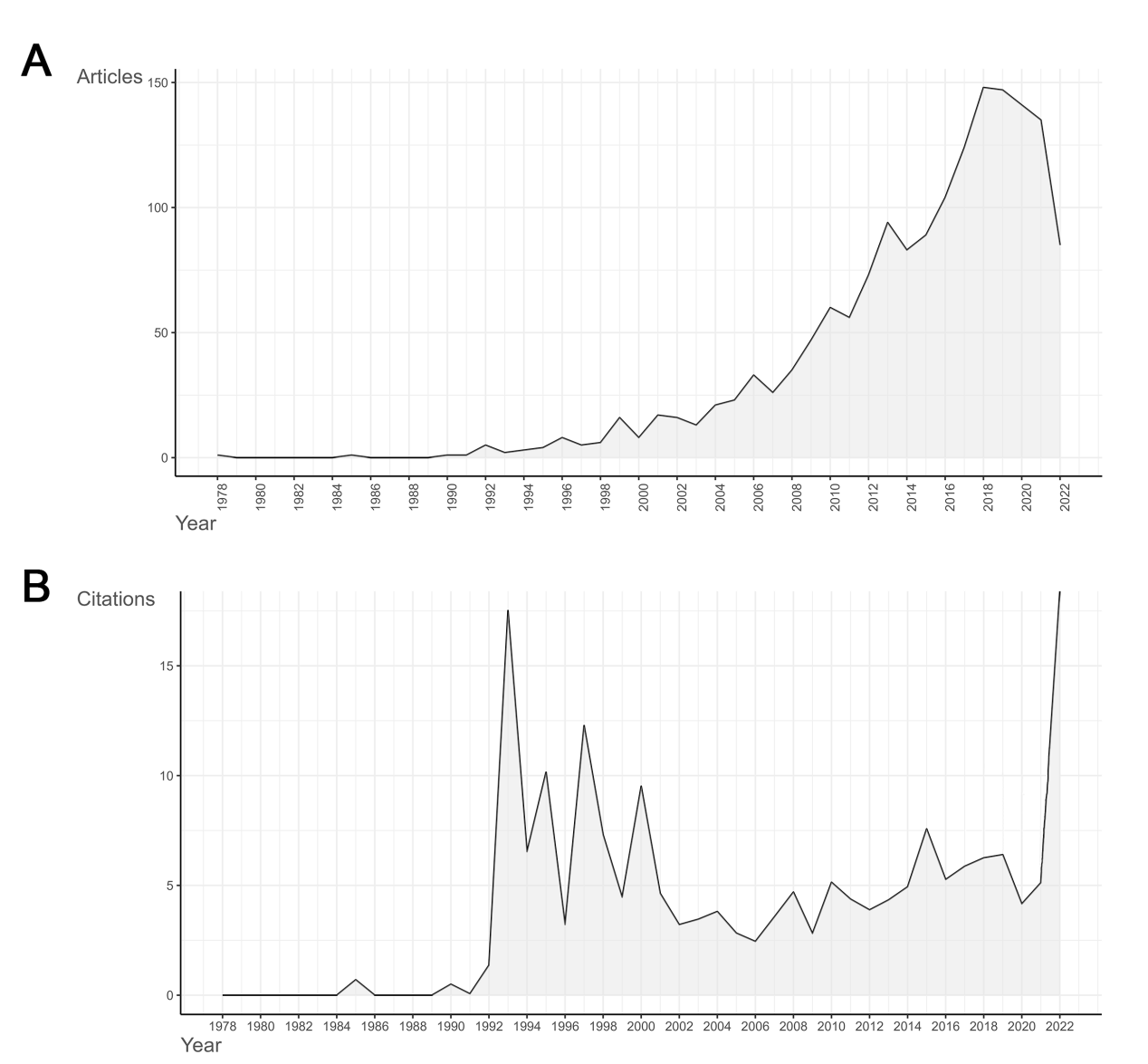


**Figure S1.** The Growth of burns associated with stem cells is arousing increasing concern.

(A) The growth of burns associated with stem cells research from 1978 to 2022.

(B) The growth trend of annual citations of burns associated with stem cells research from 1978 to 2022.


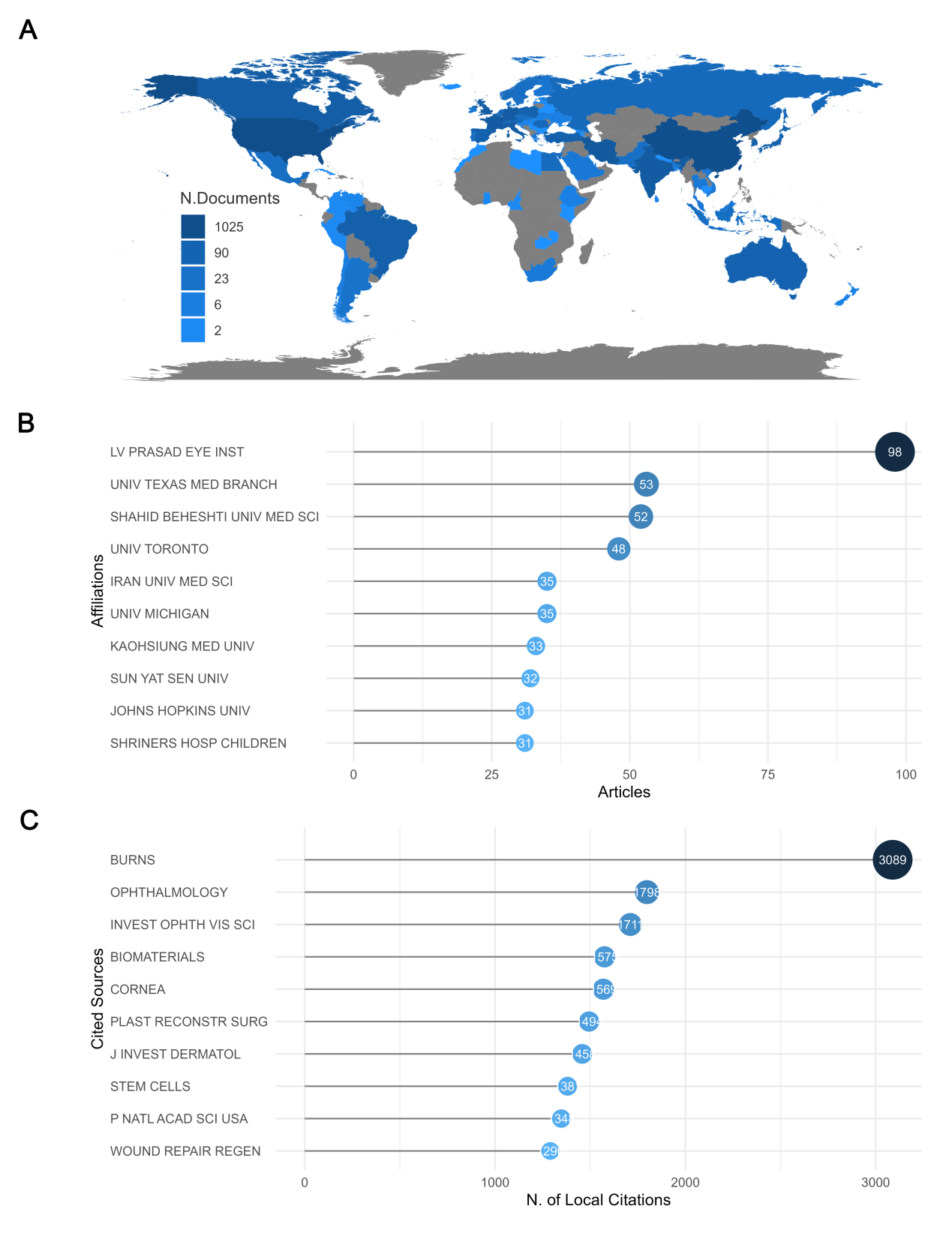


**Figure S2.** Supplementary materials for Figure 2 and Figure 3.

(A) Countries/Regions distribution world map of burns associated with stem cells research.

(B) The top 10 highest producing institutions on burns associated with stem cells research.

(C) The top 10 journals on burns associated with stem cells research with the highest number of citations.


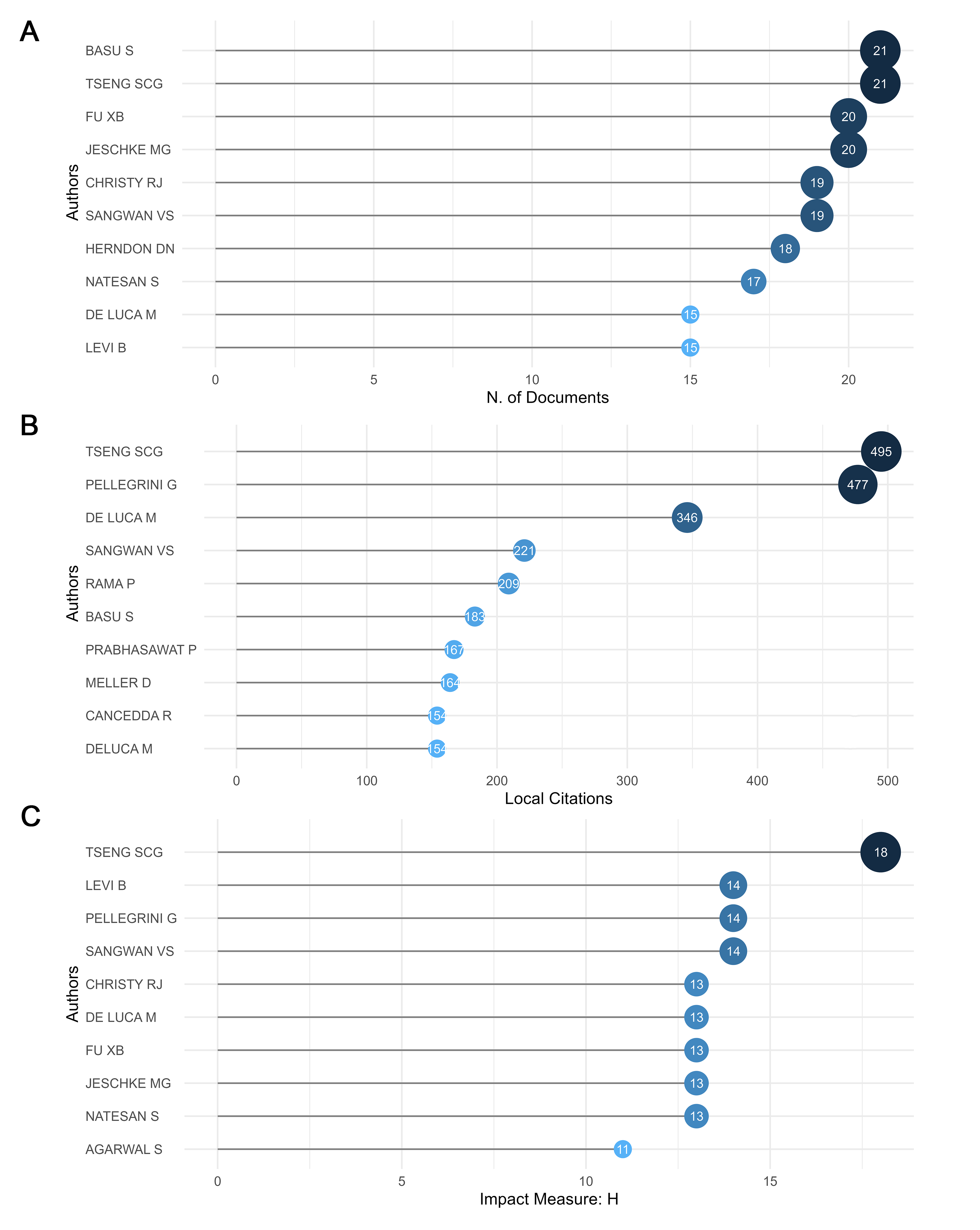


**Figure S3.** Primary authors of burns associated with stem cells research production. Woo Kyung Kim and Jonathan A. Garlick were the two most influential and contributing authors in burns associated with stem cells research.

(A) The top 10 highest producing authors on burns associated with stem cells research.

(B) The top 10 authors on burns associated with stem cells research with the highest number of citations.

(C) The top 10 authors on burns associated with stem cells research with the highest number of H-index.


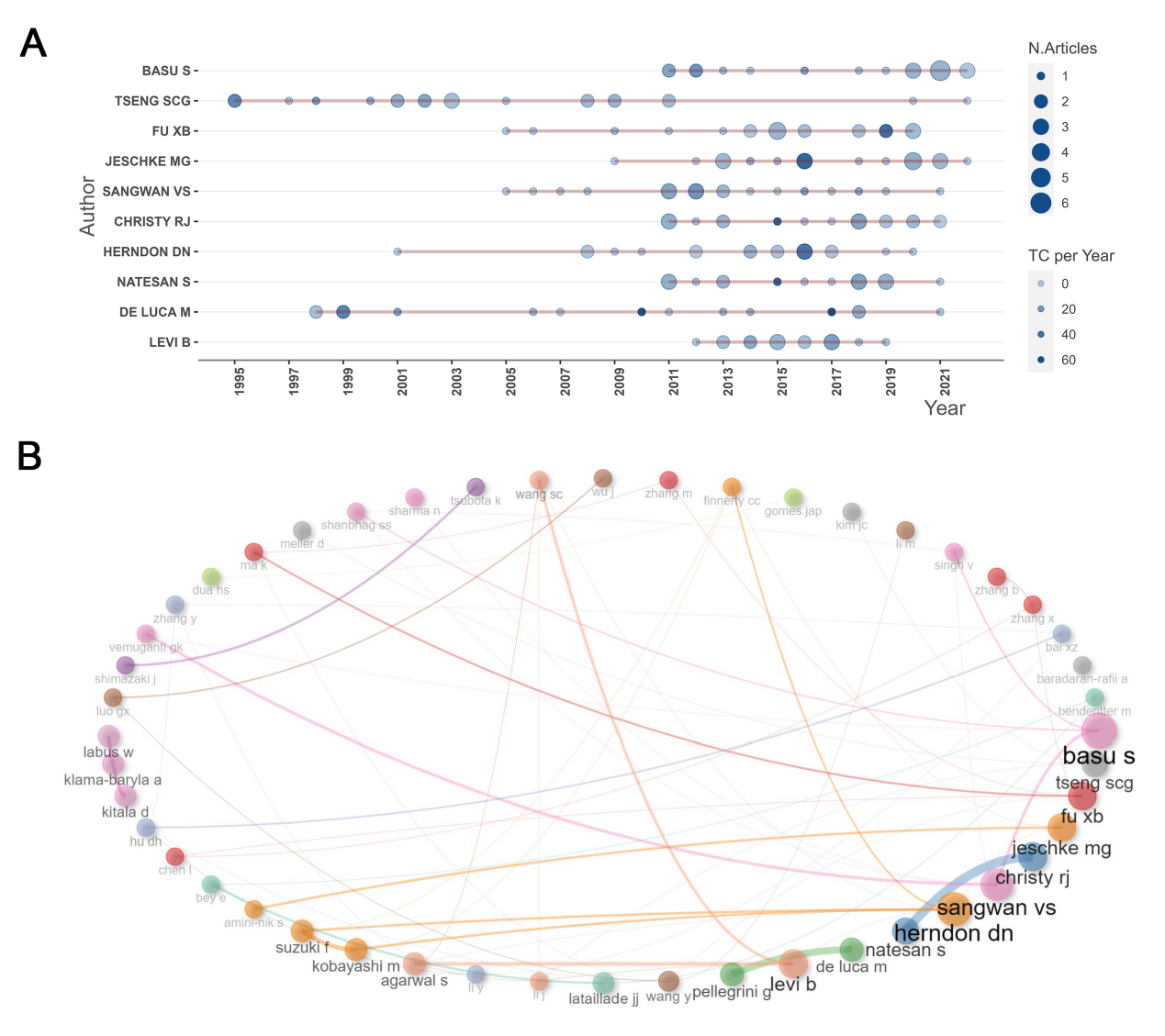


**Figure S4.** Top authors’ production over time and collaboration.

(A) A visualization network of collaboration among authors in burns associated with stem cells research.

(B) A visualization map of production over time among authors in burns associated with stem cells research.


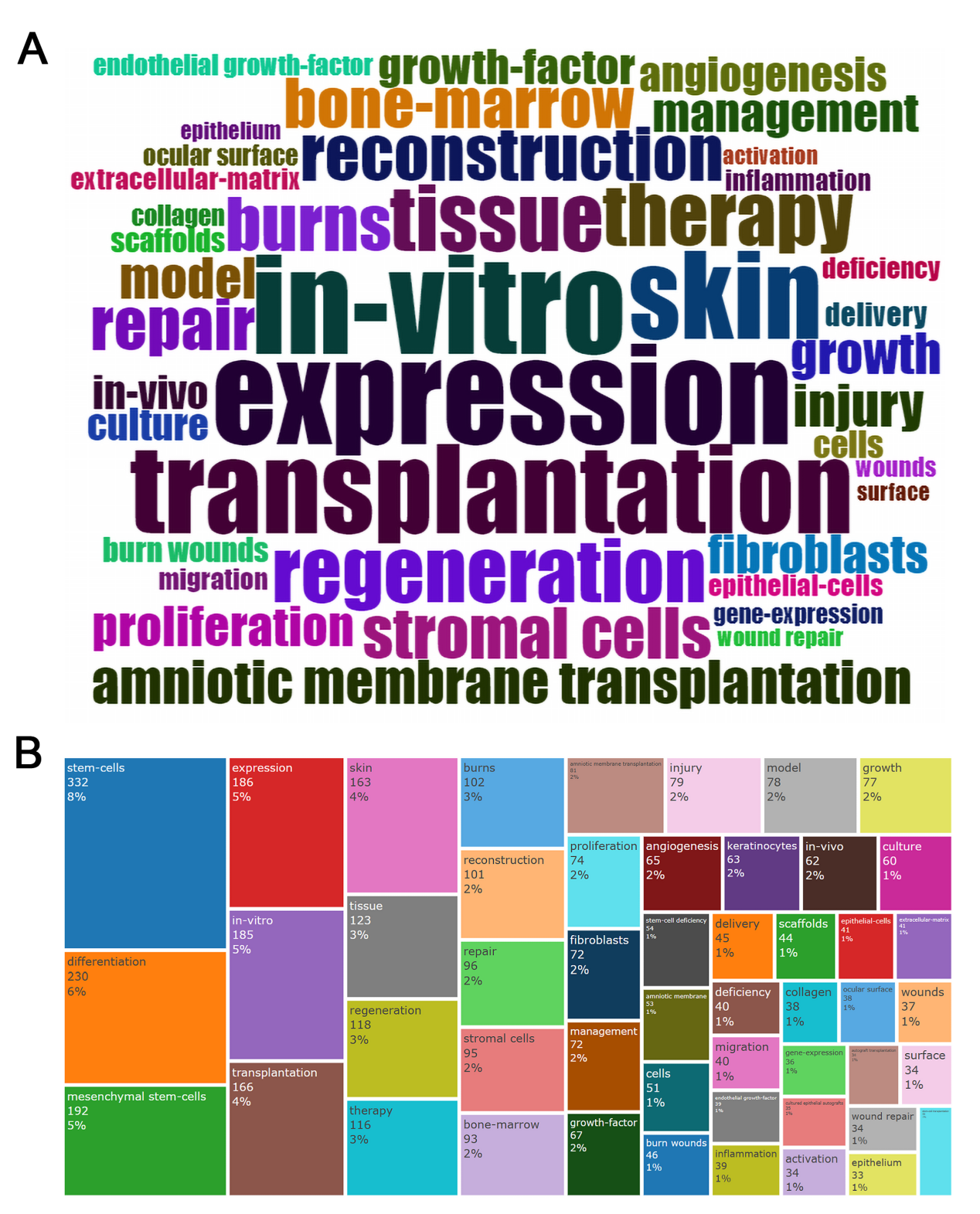


**Figure S5.** Keywords analysis.

1. Visualized tree map depends on the top 50 most frequent keywords for burns associated with stem cells research.
2. The top 10 most frequent keywords for burns associated with stem cells research.
